# Potential Microorganisms from Bronchial Lavage Fluid in Bronchiectasis Patients: Bacteria, Nontuberculous Mycobacteria, and Fungi

**DOI:** 10.2174/0118743064392945250613055623

**Published:** 2025-06-18

**Authors:** Lam Nguyen-Ho, Quoc-Khanh Tran-Le, Hoang Kim Tu Trinh, Vu Le-Thuong, Van Pham-Hung, Huong Pham-Thien, Phu Truong-Thien, Thong Dang-Vu, Dung Lam-Quoc, Ngoc Tran-Van

**Affiliations:** 1 Department of Internal Medicine, University of Medicine and Pharmacy at Ho Chi Minh City, Ho Chi Minh City, Vietnam; 2 Department of Respiratory , University Medical Center Ho Chi Minh City, Ho Chi Minh City, Vietnam; 3 Department of Respiratory , Cho Ray’s Hospital, Ho Chi Minh City, Vietnam; 4 Center for Molecular Biomedicine, University of Medicine and Pharmacy at Ho Chi Minh City, Ho Chi Minh City, Vietnam; 5 Ngoc Minh Clinic, Ho Chi Minh City, Vietnam; 6 Department of Microbiology, International Research Institute of Gene and Immunology, Ho Chi Minh City, Vietnam; 7 Department of Microbiology, Phan Chau Trinh University, Dien Ban, Vietnam; 8 Department of Microbiology , Cho Ray’s hospital, Ho Chi Minh City, Vietnam

**Keywords:** Bacteria, Bronchiectasis, Bronchial lavage fluid, Fungi, Nontuberculous mycobacteria, Pathogen

## Abstract

**Introduction:**

Bronchiectasis is a chronic lung disease characterized by irreversible bronchial dilation, often accompanied by persistent infections. Compared to sputum, the microbiological results of bronchial lavage fluid (BLF) from stable bronchiectasis patients are typically less explored. There is emerging evidence on the role of non-tuberculous mycobacteria (NTM) in the progression of bronchiectasis. This study aims to investigate the microbiological profiles of BLF and the rate of NTM detection in stable bronchiectasis patients.

**Methods:**

We conducted a prospective observational multicenter study at two endoscopy units of Cho Ray’s Hospital and University Medical Center Ho Chi Minh City, from January 2023 to January 2024. Adult patients with bronchiectasis who underwent bronchoscopy were enrolled, and the BLF was collected. The BLF samples were analyzed for bacterial and fungal pathogens using culture methods, and for NTM using the multiplex polymerase chain reaction (PCR) technique.

**Results:**

Of the 112 initially assessed patients, 99 were eligible for this study. The mean age was 63 years, and 55.6% were female. Bacterial cultures were positive in 41.9% of cases (36/86), predominantly with isolates of *Klebsiella pneumoniae* and *Pseudomonas aeruginosa*. Multi-drug resistant (MDR) *K. pneumoniae* and *Acinetobacter baumannii* were notably detected. Using PCR, NTM was detected in 52.5% of patients (52/99), predominantly slow-growing species such as *Mycobacterium xenopi* and *Mycobacterium avium-intracellulare* complex. Fungal cultures were positive in 24.6% of cases (17/69), primarily involving *Candida* spp. and *Aspergillus* spp. Patients with higher bronchiectasis severity index had higher rates of positive bacterial culture, but lower rates of NTM detection.

**Discussion:**

The high NTM detection rate in this study may be attributed to the use of BLF and sensitive molecular techniques. The frequent detection of NTM in patients with milder disease suggests that these organisms may be present in the early stages, potentially acting as an early warning for future progression. The high prevalence of MDR bacteria isolated highlights the need for revised infection control policies and tailored antibiotic strategies.

**Conclusion:**

This study demonstrated a microbial diversity in BLF, notably NTM and MDR bacteria in Vietnamese patients with bronchiectasis, emphasizing the need for routine, comprehensive microbial assessment for bronchiectasis patients. The incorporation of advanced molecular techniques can improve the detection of NTM in these patients.

## 1 INTRODUCTION

Bronchiectasis is a common chronic respiratory disorder characterized by chronic cough, daily sputum production, and/or recurrent lower airway infections [1]. While the global prevalence of bronchiectasis has been increasing, it is often underestimated. In Europe and North America, the prevalence ranges from 67 to 566 individuals per 100,000 [2], and in China, among adults aged ≥ 40, it can reach 1,200 per 100,000, posing a substantial and growing economic burden on healthcare systems [3]. The chronic bronchial infection is one of the critical aspects drive the “vicious vortex” concept of the pathophysiology of bronchiectasis [4]. Persistent infection promotes airway damage, escalating local and systemic inflammation, and impairs host immune responses. Management strategies towards this issue, particularly *Pseudomonas aeruginosa* infection, have been shown to alleviate symptoms, enhance quality of life and reduce exacerbations [5, 6].

The spectrum of infectious agents in the lower airways of bronchiectasis patients is therefore crucial, particularly in guiding future antibiotic management strategies. While most studies on the microbiological profiles have primarily used sputum samples, comparatively fewer employed bronchial lavage fluid (BLF) [7]. Though sputum sampling is non-invasive and provides valuable microbiological data, BLF obtained through bronchoscopy offers more precise targeting and minimizes contamination from oropharyngeal flora. Comparison of microbiological yield between BLF and sputum samples shows inconsistent results, with the higher yield of BLF reported by the study of Emiralioglu *et al*. and the study of Chang *et al*. [8, 9].

In addition to gram-negative and gram-positive bacteria, recent studies have also identified non-tuberculous mycobacteria (NTM) as a pathogen of structural damage and inflammation in the bronchiectasis patients’ airway. Detection rates of NTM in the lower airways of bronchiectasis patients have reached as high as 63% [10], an increase partly attributed to the use of more sensitive techniques such as nucleic acid amplification. Studies on the microbiological profiles of BLF in stable bronchiectasis patients have been lacking in Vietnam. Our study aims to address this gap. Additionally, we aim to describe the epidemiology of NTM using the multiplex polymerase chain reaction (PCR) technique.

## 2 MATERIALS AND METHODS

### 2.1 Study Design and Eligible Patients

This study was an observational, multicenter study conducted at the endoscopy units of Cho Ray’s Hospital and University Medical Center Ho Chi Minh City, from January 2023 to January 2024. Adult patients were enrolled according to the inclusion criteria as follows: (1) the patient was diagnosed with bronchiectasis based on the criteria of the British Thoracic Society guidelines 2019 [11], (2) the patient’s disease was stable, which was defined as the absence of exacerbation and no antibiotic treatments during the four weeks prior to their bronchoscopy [12], (3) the patients were given bronchoscopy for collecting BLF, and agreed to take part in this study. Exclusion criteria included retraction bronchiectasis, active pulmonary tuberculosis, or failure to undertake bronchoscopy. The study received approval from the Ethics Committee of the University of Medicine and Pharmacy at Ho Chi Minh City (528/HĐĐĐ-ĐHYD). All participants provided informed consent to take part in this study.

### 2.2 Data Collection

Following enrollment in the study, clinical data were collected for each patient, including demographic characteristics, comorbidities, clinical symptoms, radiological findings (morphology and location of bronchiectasis), forced expiratory volume in 1 second (FEV1) and forced vital capacity (FVC), and complete blood count (CBC). The neutrophil-to-lymphocyte ratio (NLR) was calculated by dividing neutrophil count by lymphocyte count. The Bronchiectasis Severity Index (BSI) including features such as age, body mass index, FEV1% predicted, hospital admission before study, exacerbations before the study, modified medical research council dyspnea scale, *Pseudomonas* colonization, colonization with other organisms, and ≥ 3 involved lobes or cystic bronchiectasis was calculated by two pulmonologists. The modified Reiff score assesses the radiological severity of bronchiectasis through the number of lobes involved and the degree of bronchial dilation (scoring 0-3 for each lobe, the patient’s lung having 6 lobes with the lingula segment considered as a lobe, and the maximum score 18) [13]. The etiologies of bronchiectasis were determined according to the European Respiratory Society guideline 2017 [1] and depended on the physician’s judgment.

### 2.3 BLF Collection

Flexible bronchoscopy was undertaken according to the conventional protocols of the University Medical Center Ho Chi Minh City and Cho Ray’s Hospital. Pre-procedure preparation included fasting for at least six hours and normal results for CBC and coagulation status. Pre-medication for bronchoscopy included subcutaneous atropine 0.5 mg and nebulizer with a combination of lidocaine 2% 2mL and salbutamol 5 mg/ 2.5 mL.

A bronchoscope (Olympus EVIS EXERA III CLV-190, Olympus Corporation, Tokyo, Japan) was inserted into the tracheobronchial tree via the nasal or oral route under local anesthesia with lidocaine 2%. The patients were monitored for both blood pressure and peripheral capillary oxygen saturation (SpO_2_) throughout the procedure. BLF was collected from the target lesion in the third bronchial segment, as previously identified on the images of chest computed tomography (CT). A 60-80 mL volume of normal saline 0.9% was instilled into the targeted bronchial branch in aliquots, and fluid recovery was facilitated using a suction kit. The collected BLF was sent to the laboratory to evaluate acid-fast bacillus (AFB) smear, bacterial and fungal culture, and detection for mycobacterium tuberculosis (MTB) and NTM.

### 2.4 Bacterial and Fungal Culture

After collection, BLF samples underwent standard microbiological analysis, starting with a Gram stain to determine initial bacterial morphology. For bacterial culture, the samples were inoculated on sheep blood agar (SBA), chocolate agar (CA), and MacConkey agar (MC). SBA and CA plates were incubated at 35-37°C in 5% CO_2_ for 16-24 hours, while MC plates were incubated under similar conditions but in a standard aerobic incubator. Following initial morphology identification, automatic identification and antibiotic susceptibility testing were performed using the Vitek 2 Compact (bioMérieux, Marcy-l'Étoile, France). For fungal culture, the samples were inoculated on Sabouraud Chloramphenicol agar and incubated at 35°C for 7 days. Following initial morphology identification, yeasts were identified automatically using the Vitek 2 Compact, and moulds were stained with Lactophenol Cotton Blue and identified under the support of a microscope.

Multidrug-resistant (MDR) bacterium was defined as a pathogen acquired non-susceptibility to at least one agent in three or more antimicrobial categories [14], including *Haemophilus influenzae* and *Achromobacter denitrificans*. Pathogens with intrinsic multi-drug resistance (*Chryseobacterium indologenes*, *Stenotrophomonas maltophilia*) were classified as MDR isolates.

### 2.5 Mycobacterial Culture and Identification

After BLF collection, an AFB smear was performed. For culture, 0.5 mL BLF was inoculated into a BACTEC MGIT 960 tube (BD, Sparks, USA) and 0.2 mL onto two solid Lowenstein-Jensen (LJ) slants. MGIT tubes were incubated at 37 °C for up to 42 days and LJ slants were examined weekly for 8 weeks. Any positive MGIT growth or LJ colonies were confirmed as AFB by Ziehl–Neelsen staining and PCR for MTB; cultures without growth by the end of incubation were reported negative.

### 2.6 Molecular Detection and Identification of NTM and MTB

#### 2.6.1 Primer and Probes Design for Detection of NTM and MTB

The target for primers and TaqMan probes was the ribosomal 16S rRNA. Sequences of 16S rRNA for mycobacterium species were obtained from the National Center for Biotechnology Information (NCBI) gene database. Using BioEdit software (BioEdit v7.2.5, Ibis Biosciences, Carlsbad, CA), specific sequences for the mycobacteria species of interest were identified. These were then used to develop five multiplex real-time PCR assays (**S1** and Table **S1**)

### 2.7 DNA Extraction and PCR Assay Protocol

DNA was then extracted from BLF samples using ^NK^DNARNAprep-MAGBEAD kit (Nam Khoa Company, Ho Chi Minh City, Vietnam), employing the KingFisher system (Thermo Fisher Scientific, Waltham, MA, USA). This kit includes dedicated binding, washing, and elution buffers and follows a standardized five-step automated protocol that ensures consistent quality across extractions. The method has been validated for its efficiency and sensitivity across a range of clinical samples and pathogens, demonstrating non-inferior performance compared to commercial kits such as MagnaPure (Roche), QIAGEN, BOOM, and Trizol-LS.

5 µL of DNA extraction samples were added to 15 µL of each reaction mixture. To detect potential false negatives, we employed internal control, including the 16S rRNA gene for microbial presence. The PCR assays were performed using a CFX96 Real-Time PCR Detection System (Bio-Rad Laboratories, Hercules, CA, USA). The thermal cycling protocol began with DNA denaturation at 96°C for 10 minutes, followed by 35 cycles of denaturation at 98°C and incubating at 60°C for 30 seconds each, ending with a hold at 10°C.

To ensure contamination control, in addition to performing bronchoscopy strictly under institutional protocol, DNA extraction and PCR setup were carried out in physically separated pre- and post-PCR rooms. Work surfaces and equipment were disinfected and UV-irradiated before and after each session. Staff used aerosol-resistant filter tips, strict aseptic technique, and protective gloves. Every run included negative controls; any suspected assay was discarded and repeated. After amplification, samples were assessed for sensitivity, and positives were confirmed when all fluorescent channels reached a cycle threshold below 35.

### 2.8 Statistical Analysis

Based on Fujita *et al*. [15], who reported an NTM detection rate of approximately 47.8% in bronchiectasis patients, we calculated that enrolling 96 participants

would allow estimation of this prevalence with a ±10% margin of error at the 95% confidence level. We ultimately enrolled 99 patients, thereby satisfying this requirement. The normality of the data was evaluated using histograms and the Kolmogorov-Smirnov test. Means (± SD, standard deviation) were used to present normally distributed data, whereas medians (IQR, interquartile range) were used for data that were not normally distributed. Proportions or percentages describe categorical data. For normally distributed variables, the t-test was employed, and the Mann-Whitney U test was applied to variables that were not normally distributed. A two-sided p-value of less than 0.05 was considered statistically significant. SPSS statistical software (version 22.0, IBM Corp, Armonk, NY, 2017) was used to process the data.

## 3 RESULTS

### 3.1 Demographic and Clinical Characteristics of Study Subjects

**Fig. (1):**
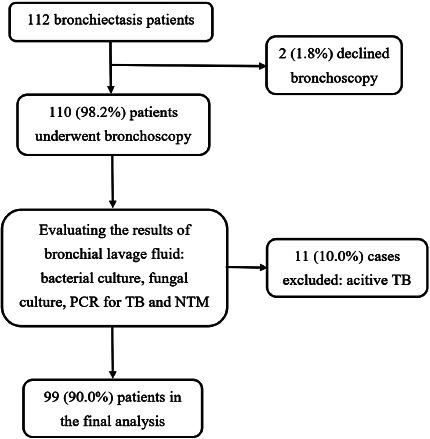
A flow chart to present the process of enrollment.

Between January 2023 and January 2024, 112 patients were eligible for our study. Of these, 2 refused to participate and 11 were excluded after *Mycobacterium tuberculosis* was detected in the BLF specimens (either by AFB smear, PCR, or culture for tuberculosis), leaving a total of 99 cases included in the final analysis (Fig. **1**). The mean age was 63 ± 12 years, with 60.6% of patients being over 60 years old. Females were slightly predominant, comprising 55.6% of the cases. Detailed demographic and clinical data are presented in Table **1**. All cases had multiplex PCR for NTM and MTB detection in BLF, but bacterial and fungal cultures were performed on 86.9% (86/99) and 69.7% (69/99) of cases, respectively, depending on the physician’s judgement. NLR showed an association with the severity of bronchiectasis defined by BSI (r = 0.373, p = 0.001).

### 3.2 Bacterial Profiles from BLF of Bronchiectasis Patients

Positive bacterial cultures were identified in 36 (41.9%) cases, with the frequency distribution of organisms detailed in Fig. (**2**). The most frequent isolates were *Klebsiella pneumoniae* (28.2%) and *P. aeruginosa* (25.6%). Polymicrobial isolations were observed in three cases: *K. pneumoniae + H. influenzae, Staphylococcus aureus + H. influenzae,* and *Stenotrophomonas maltophilia + Chryseobacterium indologenes*. Patients with dry cough had higher bacterial yields (p = 0.013). Additionally, older patients and those with higher BSI also showed higher positive culture (Table **2**).

MDR bacteria were determined in 35.9% (14/39) of the isolated organisms. Table **S2** describes antimicrobial resistance profiles of bacteria isolated from BLF. Patients with MDR isolate had higher BSI scores but no statistical significance (6.0 [3.5 – 11.0] *vs.* 4.0 [3.0 – 6.0], p = 0.123) and significantly higher episodes of hospitalization in the previous year (76.9% *vs.* 16.7%, p < 0.001) than those with susceptible isolates.

### 3.3 Non-tuberculous Mycobacteria in Patients with Bronchiectasis

NTM was found in 52.5% of patients, with 90.4% being slowly growing species. Patients with frequent expectoration were more likely to have NTM detected than those in the dry cough group (p = 0.029). There were six cases with two NTM species to be simultaneously detected (two cases with *M. avium-intracellulare complex* + *M. szulgai*, one case with *M. avium-intracellulare complex* + *M. xenopi*, two cases with *M. szulgai* + *M. tilburgii*, one case with *M. abscessus complex* + *M. chelonae*). Distribution of NTM species was presented in Fig. (**3**), in which *M. xenopi* and *Mycobacterium avium-intracellulare complex* (MAC) were the most common. Patients with lower BSI score had a significantly higher frequency of NTM detection (Table **3**).

### 3.4 Fungal Characteristics in BLF from Bronchiectasis Patients

Among the 69 BLF samples for fungal culture were performed, fungal species were detected in 17 cases (14 cases with *Candida spp*, one case with both *Candida spp* and *Penicillium spp*, and two cases with *Aspergillus spp*). Fungi were detected more frequently in cases with greater bronchial dilation on chest CT according to the modified Reiff score (3.0 [2.0 – 5.0] *vs.* 2.0 [1.3 – 4.0], p = 0.044).

## 4 DISCUSSION

Our study highlighted the microbial diversity in BLF from bronchiectasis patients in Vietnam, including bacteria, fungi, and NTM. Characteristics of bronchiectasis patients in our study showed similarities to previous studies [3, 16], except the predominance of both mild bronchiectasis (60.6%) and cylindrical pattern on chest CT images (64.6%). Most previous studies used sputum to detect microorganisms in stable bronchiectasis patients; meanwhile, our study employed BLF, which could reflect microorganisms in the distant airways more effectively. Our study also showed that the simple NLR was associated with the severity of bronchiectasis based on the BSI score. This finding was similar to a previous study conducted in the Spanish population [17].

### 4.1 Bacterial species

In our study, *P. aeruginosa* and *H. influenzae* remained the frequently detected organisms in patients with stable bronchiectasis, consistent with findings from previous studies [18, 19]. These organisms were associated with more severe disease and a greater incidence of exacerbation [2, 20]. The antimicrobial resistance of these bacteria, a great concern, can develop in the follow-up study of Wagner *et al*. due to antibiotic therapy and chronic bacterial infection [21]. However, they were still susceptible in our observational study, and one of the reasons could be that the majority of mild bronchiectasis cases were accounted for in our cohort.

*K. pneumoniae* emerged as the most common bacterium detected in BLF of our bronchiectasis patients, which was similar to the Indian bronchiectasis cohort [22]. Understanding its role in bronchiectasis patients still needs to be improved. Therefore, in-depth studies about the effects of *K. pneumoniae* in bronchiectasis patients is required. Moreover, *K. pneumoniae* resistant to carbapenems and fluoroquinolones was common in our study, along with another unusual bacterium (*Acinetobacter baumannii*). This finding should be interpreted in the context of nosocomial bacteria in a country with a high prevalence of antimicrobial resistance, such as Vietnam. Notably, 35.9% of isolated bacteria in BLF had antimicrobial resistance. These isolations may originate in hospital settings, as evidenced by the fact that these patients experience significantly more frequent exacerbations requiring hospitalization, which aligns with the previous study [23]. It is uncertain whether bronchiectasis patients with MDR bacteria have worsening long-term clinical outcomes. The high isolated rate of MDR strains indicates a need to revise infection control policies, especially by enhancing hygiene and disinfection protocols, selecting the appropriate strategy for antibiotic therapy (based on the antimicrobial susceptibility test, the preferred option of inhaled/nebulized antibiotics with a lower resistance rate) [23], and even propose the strict criteria of hospitalization for bronchiectasis patients in Vietnam.

### 4.2 NTM Detection

The globally estimated prevalence of NTM infection in bronchiectasis patients is about 10% [24], and this prevalence in the Asian population is 9.5% (4.6% - 18.7%) [25]. However, this rate varies due to multiple factors, including differences in microbiological detection methods, the practice preferences of each medical center regarding mycobacterial culture indications (routine testing *vs.* testing based on clinical suspicion), the types of respiratory samples used for analysis, and geographical influences [10, 26-28]. The NTM detection rate in bronchiectasis patients was higher in the United States at 63% (1158/1826) and on the rising trend in European and Asian countries in recent years. The study by Suska *et al*. in 2022 conducted in Italy had a prevalence of 26.1% (the previous rate 12.2%) [10, 29]. Using multiplex PCR technique and BLF, our study demonstrated that NTM is commonly found in Vietnamese patients with bronchiectasis (52.5%), likewise a study in Japan employing BLF samples and another study in China using newer microbiology detection techniques with MPB64 antigen have reported higher detection rates (47.8% and 61.2%, respectively) [15, 30].

MAC and *M. xenopi* were the most common NTM species in our study. The distribution of NTM species changes according to geographic features, and MAC was documented as the most frequently detected mycobacteria in bronchiectasis patients, especially in the United States [10, 31-33]. On the other hand, *M. xenopi* was reported more commonly in Western European countries. However, NTM studies in Northern Vietnam and Cambodia revealed no *M. xenopi* detected [34, 35]. This finding could imply that the distribution of NTM species varies between regions in Vietnam.

The clinical implications of NTM detection in bronchiectasis patients remain a matter of active investigation, with mounting evidence suggesting that NTM may play a pathogenic role beyond mere colonization. Fujita *et al*. conducted a prospective cohort study comparing frailty in patients with NTM lung disease and bronchiectasis, demonstrating that those with NTM infection exhibited significantly higher frailty indices and worse functional outcomes, implicating NTM in disease progression and poorer clinical prognosis [15]. In our study, NTM was more frequently detected in milder cases of bronchiectasis, as measured by the BSI. This finding contrasts with the study from Italy, in which patients had the more severe disease and immunodeficiency as a common etiology of bronchiectasis [29]. This difference could result from discrepancies in the study population, the detection method of NTM, and the type of specimen employed. Our study used BLF via bronchoscopy, which can lead to more cases of milder bronchiectasis (dry cough and local bronchiectasis) being enrolled. The presence of NTM in mild patients suggests that NTM can exist from the earlier stages of the disease, potentially triggering airway inflammation and damage that later leads to more severe bronchiectasis. Detecting NTM in mild disease may serve as an “early warning” prompting closer monitoring of lung function, frailty, and quality of life. Routine BLF screening for NTM, regardless of disease stage, could enable earlier diagnosis and timely, species-specific multidrug therapy. Adding longitudinal follow-up with frailty assessments, serial imaging, and biomarkers can help distinguish true NTM disease from benign colonization and gauge the impact of treatment on exacerbations and radiological progression.

### 4.3 Fungal Profiles

Our findings also revealed that *Candida spp* and *Aspergillus spp* were the most frequently isolated fungal species. This aligns with results from other studies [31, 36] but did not show the association between previous pulmonary tuberculosis and *Aspergillus spp* detection, which was mentioned in the study of Yang *et al*. [37]. The study of Cheng *et al*. revealed that the Reiff score can be useful in predicting disease severity and prognosis of bronchiectasis patients [38]. Moreover, our study described the positive fungal culture existing in more severe bronchiectasis on radiography, as defined by higher modified Reiff scores. However, it remains unclear whether these fungi merely colonize the airways or contribute to bronchiectasis progression or trigger exacerbations. Further studies are needed to explore their role in bronchiectasis.

## 5 STUDY LIMITATION

Our study has several limitations. First, the limited sample size prevented a detailed analysis. Second, bacterial and fungal cultures of BLF were conducted at the discretion of the physician rather than uniformly across the entire study sample. However, this approach reflects real-world practice in limited-resource settings like Vietnam, where tuberculosis diagnostics often take precedence with less concern for bacteria and fungi in patients with stable bronchiectasis. In addition, we did not analyze which subjects with NTM isolation have NTM pulmonary disease. We applied homogenized and strict protocols to collect BLF and to detect NTM in BLF, but the minimal contamination issue remains unavoidable. Finally, the microbiological results may reflect the local microbiological etiology in bronchiectasis, specific to Southern Vietnam, which may not represent the significant geographical diversity.

## CONCLUSION

Our study on Vietnamese patients with bronchiectasis reveals a complex microbiological environment, characterized by a high prevalence of NTM and MDR bacteria in BLF. It remains unclear whether these microorganisms are causative factors in developing bronchiectasis or simply colonizing patients with pre-existing bronchiectasis. However, patients harbouring these microorganisms exhibited greater severity of bronchiectasis, except for NTM detection. This suggests that tailored treatment strategies targeting specific microbial profiles may improve patient outcomes. Additionally, our study demonstrates that using advanced molecular techniques improves the detection of NTM. This research contributes to a broader understanding of the microbiology of bronchiectasis and underscores the need for further studies to refine diagnostic protocols applying nucleic acid amplification techniques.

## Figures and Tables

**Fig. (2):**
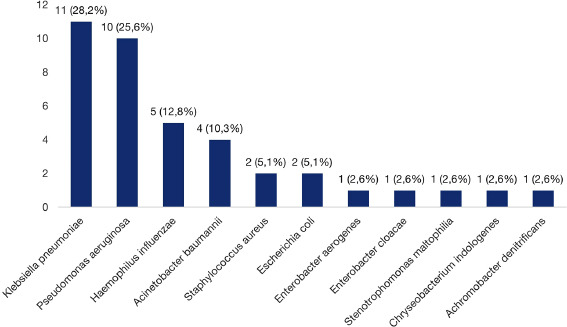
Distribution of bacteria isolated from bronchial lavage fluid of bronchiectasis patients.

**Fig. (3):**
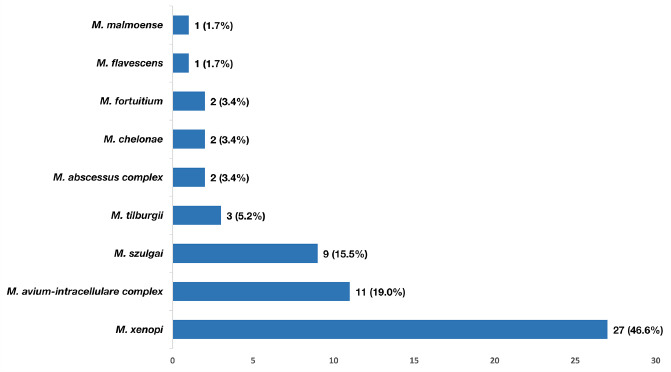
Frequency of nontuberculous mycobacteria species detected from bronchial lavage fluid of bronchiectasis patients using multiplex polymerase chain reaction technique.

**Table 1:** Clinical characteristics of bronchiectasis patients (n = 99).

**Characteristics**	**Total (n = 99)**
**Age** (mean ± SD)	63 ± 12
**Female gender** (n, %)	55 (55.6)
**Body mass index** (kg/m^2^) (mean ± SD)	21.1 ± 3.1
**Symptoms**	-
Expectoration (n, %)	80 (80.8)
Sputum volume (mL) (Median [IQR])	5.0 [5.0 – 10.0]
Dyspnea (n, %)	41 (41.4)
**History of hemoptysis** (n, %)	29 (29.3)
**Past year history of exacerbation** (n, %)	15 (15.2)
**Comorbidities** (n, %)	-
Hypertension	22 (22.2)
Diabetes mellitus	10 (10.1)
Cirrhosis	2 (2.0)
Chronic renal disease	3 (3.0)
Anxiety - depression	2 (2.0)
**Laboratory findings** (median, IQR)	-
WBC (K/mm^3^)	7.9 [6.7 – 10.1]
NLR	2.8 [1.7 – 4.0]
Platelets (K/mm^3^)	280.0 [227.0 – 341.0]
FEV1%	75.0 [65.8 – 81.0]
FVC%	76.5 [69.3 – 80.1]
**Dominant radiological pattern** (n, %)	-
Cylindrical	64 (64.6)
Varicose	13 (13.1)
Cystic	11 (11.1)
Combination	11 (11.1)
**Bronchiectasis distribution** (n, %)	-
Upper lobe	66 (66.7)
Middle lobe	51 (51.5)
Lower lobe	56 (56.6)
Bilateral	56 (56.6)
**Modified Reiff score** (median, IQR)	3.0 [2.0 – 4.0]
**Bronchiectasis etiology** (n, %)	-
Post tuberculosis	24 (24.3)
Immunodeficiency	5 (5.1)
Asthma	3 (3.0)
COPD	1 (1.0)
NTM infection	29 (29.3)
Other etiologies (post pulmonary infections, eosinophil pneumonitis, *e.g.*)	4 (4.0)
Unknown	33 (33.3)
**Bronchiectasis Severity Index** (n, %)	-
Mild (0-4)	60 (60.6)
Moderate (5-8)	25 (25.3)
Severe (≥ 9)	14 (14.1)

**Note:** COPD: Chronic obstructive pulmonary disease; FEV1: Forced expiratory volume in 1 second; FVC: Forced vital capacity; IQR: Interquartile range; NLR: Neutrophil to lymphocyte ratio; NTM: Non-tuberculous mycobacteria; SD: Standard deviation; WBC: White blood cells

**Table 2:** Comparison of clinical characteristics between negative and positive bacterial-culture groups (n = 86).

**Characteristics**	**Positive Bacterial Culture (n = 36)**	**Negative Bacterial Culture (n = 50)**	*p-value*
**Age** (mean ± SD)	65.0 (59.5 -71.8)	60.5 (53.0 – 67.0)	**0.023^!^**
**Female gender** (n, %)	21 (58.3)	26 (52.0)	0.561^$^
**WBC** (K/mm^3^) (Median, IQR)	7.9 [6.9 – 9.9]	7.9 [6.7 – 10.4]	0.859^!^
**NLR** (Median, IQR)	2.8 [1.7 – 3.5]	2.7 [1.7 – 4.1]	0.869^!^
**Modified Reiff score** (median, IQR)	3.0 [2.0 – 5.0]	2.0 [2.0 – 4.0]	0.109^!^
**Bronchiectasis Severity Index** (median, IQR)	5.0 [3.0 – 7.5]	4.0 [2.0 – 6.0]	**0.048^!^**

^!^Mann-Whitney U test; ^$^Chi-Square test IQR: Interquartile range; NLR: Neutrophil to lymphocyte ratio; SD: Standard deviation; WBC: White blood cells

**Table 3:** Comparison of clinical characteristics between negative and positive NTM detection groups by multiplex polymerase chain reaction in bronchial lavage fluid (n = 99).

-	**NTM Positive** (n = 52)	**NTM Negative** (n = 47)	*p-value*
**Age** (mean ± SD)	59.8 ± 11.6	64.3 ± 11.8	**0.023^!^**
**Female gender** (n, %)	27 (51.9)	28 (59.6)	0.561^$^
**WBC** (K/mm^3^) (median, IQR)	21.0 (18.4 – 22.5)	21.8 (19.2 – 23.4)	0.859^!^
**NLR** (median, IQR)	7.7 (6.7 – 9.6)	8.8 (6.8 – 10.5)	0.869^!^
**Middle lobe distribution** (n, %)	23 (44.2)	28 (59.6)	0.127^$^
**Modified Reiff score** (median, IQR)	2.0 (2.0 – 4.0)	3.0 (2.0 – 5.0)	0.109^!^
**Bronchiectasis Severity Index** (median, IQR)	3.0 (2.0 – 5.0)	4.0 (3.0 – 8.0)	**0.048^!^**

^!^Mann-Whitney U test; ^$^Chi-Square test IQR: Interquartile range; NLR: Neutrophil-to-lymphocyte ratio; NTM: Nontuberculous mycobacteria; SD: Standard deviation; WBC: White blood cells.

## Data Availability

The data supporting the findings of the article is available in the Zenodo Repository at https://zenodo.org/records/15700168, reference number 10.5281/zenodo. 15700168.

## LIST OF ABBREVIATIONS

BLF: = Bronchial Lavage Fluid
BSI: = Bronchiectasis Severity Index
CT: = Computed Tomography
FEV1: = Forced Expiratory Volume in 1 Second
LJ: = Lowenstein-Jensen
MDR: = Multidrug Resistant
MTB: = Mycobacterium Tuberculosis
NTM: = Nontuberculous Mycobacteria
PCR: = Polymerase Chain Reaction

## STANDARD OF REPORTING

STROBE guidelines were followed.

## References

[1] Polverino E., Goeminne P.C., McDonnell M.J., Aliberti S., Marshall S.E., Loebinger M.R., Murris M., Cantón R., Torres A., Dimakou K., De Soyza A., Hill A.T., Haworth C.S., Vendrell M., Ringshausen F.C., Subotic D., Wilson R., Vilaró J., Stallberg B., Welte T., Rohde G., Blasi F., Elborn S., Almagro M., Timothy A., Ruddy T., Tonia T., Rigau D., Chalmers J.D. (2017). European Respiratory Society guidelines for the management of adult bronchiectasis.. Eur. Respir. J..

[2] Guan W., Han X., de la Rosa-Carrillo D., Martinez-Garcia M.A. (2019). The significant global economic burden of bronchiectasis: A pending matter.. Eur. Respir. J..

[3] Roberts J.M., Goyal V., Kularatna S., Chang A.B., Kapur N., Chalmers J.D., Goeminne P.C., Hernandez F., Marchant J.M., McPhail S.M. (2023). The economic burden of bronchiectasis.. Chest.

[4] Chalmers J.D., Hill A.T. (2013). Mechanisms of immune dysfunction and bacterial persistence in non-cystic fibrosis bronchiectasis.. Mol. Immunol..

[5] Guan W., Xu J., Luo H., Xu X., Song Y., Ma W., Liang Z., Liu X., Zhang G., Zhang X., Li R., Zhu S., Zhang Y., Cai X., Wei L., Tian D., Zhao H., Chen P., Qu J., Zhong N., TORNASOL Study Group (2023). A double-blind randomized placebo-controlled phase 3 trial of tobramycin inhalation solution in adults with bronchiectasis with pseudomonas aeruginosa infection.. Chest.

[6] Rogers G.B., Bruce K.D., Martin M.L., Burr L.D., Serisier D.J. (2014). The effect of long-term macrolide treatment on respiratory microbiota composition in non-cystic fibrosis bronchiectasis: An analysis from the randomised, double-blind, placebo-controlled BLESS trial.. Lancet Respir. Med..

[7] Miao X.Y., Ji X.B., Lu H.W., Yang J.W., Xu J.F. (2015). Distribution of major pathogens from sputum and bronchoalveolar lavage fluid in patients with noncystic fibrosis bronchiectasis.. Chin. Med. J. (Engl.).

[8] Emiralioglu N., Sancak B., Tugcu G.D., Sener B., Yalcın E., Dogru D., Kiper N., Ozcelik U. (2017). Comparison of Bronchoalveolar Lavage and Sputum Microbiology in Patients with Primary Ciliary Dyskinesia.. Pediatr. Allergy Immunol. Pulmonol..

[9] Chang A.B., Boyce N.C., Masters I.B., Torzillo P.J., Masel J.P. (2002). Bronchoscopic findings in children with non-cystic fibrosis chronic suppurative lung disease.. Thorax.

[10] Aksamit T.R., O’Donnell A.E., Barker A., Olivier K.N., Winthrop K.L., Daniels M.L.A., Johnson M., Eden E., Griffith D., Knowles M., Metersky M., Salathe M., Thomashow B., Tino G., Turino G., Carretta B., Daley C.L., Bronchiectasis Research Registry Consortium (2017). Adult Patients With Bronchiectasis.. Chest.

[11] T Hill A., L Sullivan A., D Chalmers J., De Soyza A., Stuart Elborn J., Andres Floto R., Grillo L., Gruffydd-Jones K., Harvey A., S Haworth C., Hiscocks E., R Hurst J., Johnson C., Peter Kelleher W., Bedi P., Payne K., Saleh H., J Screaton N., Smith M., Tunney M., Whitters D., Wilson R., R Loebinger M. (2019). British thoracic society guideline for bronchiectasis in adults.. Thorax.

[12] Hill A.T., Haworth C.S., Aliberti S., Barker A., Blasi F., Boersma W., Chalmers J.D., De Soyza A., Dimakou K., Elborn J.S., Feldman C., Flume P., Goeminne P.C., Loebinger M.R., Menendez R., Morgan L., Murris M., Polverino E., Quittner A., Ringshausen F.C., Tino G., Torres A., Vendrell M., Welte T., Wilson R., Wong C., O’Donnell A., Aksamit T., EMBARC/BRR definitions working group (2017). Pulmonary exacerbation in adults with bronchiectasis: A consensus definition for clinical research.. Eur. Respir. J..

[13] Reiff D.B., Wells A.U., Carr D.H., Cole P.J., Hansell D.M. (1995). CT findings in bronchiectasis: Limited value in distinguishing between idiopathic and specific types.. AJR Am. J. Roentgenol..

[14] Magiorakos A.P., Srinivasan A., Carey R.B., Carmeli Y., Falagas M.E., Giske C.G., Harbarth S., Hindler J.F., Kahlmeter G., Olsson-Liljequist B., Paterson D.L., Rice L.B., Stelling J., Struelens M.J., Vatopoulos A., Weber J.T., Monnet D.L. (2012). Multidrug-resistant, extensively drug-resistant and pandrug-resistant bacteria: An international expert proposal for interim standard definitions for acquired resistance.. Clin. Microbiol. Infect..

[15] Fujita K., Ito Y., Yamamoto Y., Kanai O., Imakita T., Oi I., Ito T., Saito Z., Mio T. (2022). Comparison of frailty in patients with nontuberculous mycobacterial lung disease and bronchiectasis: A prospective cohort study.. BMC Pulm. Med..

[16] Dhar R., Singh S., Talwar D., Mohan M., Tripathi S.K., Swarnakar R., Trivedi S., Rajagopala S., D’Souza G., Padmanabhan A., Baburao A., Mahesh P.A., Ghewade B., Nair G., Jindal A., Jayadevappa G.D.H., Sawhney H., Sarmah K.R., Saha K., Anantharaj S., Khanna A., Gami S., Shah A., Shah A., Dutt N., Garg H., Vyas S., Venugopal K., Prasad R., Aleemuddin N.M., Karmakar S., Singh V., Jindal S.K., Sharma S., Prajapat D., Chandrashekaria S., McDonnell M.J., Mishra A., Rutherford R., Ramanathan R.P., Goeminne P.C., Vasudev P., Dimakou K., Crichton M.L., Jayaraj B.S., Kungwani R., Das A., Sawhney M., Polverino E., Torres A., Gulecha N.S., Shteinberg M., De Soyza A., Mangala A., Shah P., Chauhan N.K., Jajodia N., Singhal A., Batra S., Hasan A., Limaye S., Salvi S., Aliberti S., Chalmers J.D. (2019). Bronchiectasis in India: Results from the European Multicentre Bronchiectasis Audit and Research Collaboration (EMBARC) and Respiratory Research Network of India Registry.. Lancet Glob. Health.

[17] Martinez-García M.Á., Olveira C., Girón R., García-Clemente M., Máiz-Carro L., Sibila O., Golpe R., Méndez R., Rodríguez Hermosa J.L., Barreiro E., Prados C., Rodríguez López J., de la Rosa D. (2022). Peripheral neutrophil-to-lymphocyte ratio in bronchiectasis: A marker of disease severity.. Biomolecules.

[18] Borekci S., Halis A., Aygun G., Musellim B. (2016). Bacterial colonization and associated factors in patients with bronchiectasis.. Ann. Thorac. Med..

[19] Dimakou K., Triantafillidou C., Toumbis M., Tsikritsaki K., Malagari K., Bakakos P. (2016). Non CF-bronchiectasis: Aetiologic approach, clinical, radiological, microbiological and functional profile in 277 patients.. Respir. Med..

[20] Yang S.H., Song M.J., Kim Y.W., Kwon B.S., Lim S.Y., Lee Y.J., Park J.S., Cho Y.J., Lee J.H., Lee C.T., Kim H.J. (2024). Understanding the effects of Haemophilus influenzae colonization on bronchiectasis: A retrospective cohort study.. BMC Pulm. Med..

[21] Wagner M.J., Dimitrov M., Lam G.Y., Leung W., Tyrrell G.J., Vethanayagam D. (2023). Microbiology sampling in non-cystic fibrosis bronchiectasis cases from northern Alberta.. PLoS One.

[22] Dhar R., Singh S., Talwar D., Murali Mohan B.V., Tripathi S.K., Swarnakar R., Trivedi S., Rajagopala S., D’Souza G., Padmanabhan A., Archana B., Mahesh P.A., Ghewade B., Nair G., Jindal A., Jayadevappa G.D.H., Sawhney H., Sarmah K.R., Saha K., Anantharaj S., Khanna A., Gami S., Shah A., Shah A., Dutt N., Garg H., Vyas S., Venugopal K., Prasad R., Aleemuddin N.M., Karmakar S., Singh V., Jindal S.K., Sharma S., Prajapat D., Chandrashekar S., Loebinger M., Mishra A., Blasi F., Ramanathan R.P., Goeminne P.C., Vasudev P., Shoemark A., Jayaraj B.S., Kungwani R., Das A., Sawhney M., Polverino E., Welte T., Gulecha N.S., Shteinberg M., Mangala A., Shah P., Chauhan N.K., Jajodia N., Singhal A., Batra S., Hasan A., Aliberti S., Crichton M.L., Limaye S., Salvi S., Chalmers J.D., EMBARC-India study group (2023). Clinical outcomes of bronchiectasis in India: Data from the EMBARC/Respiratory Research Network of India registry.. Eur. Respir. J..

[23] Inchingolo R., Pierandrei C., Montemurro G., Smargiassi A., Lohmeyer F.M., Rizzi A. (2021). Antimicrobial resistance in common respiratory pathogens of chronic bronchiectasis patients: A literature review.. Antibiotics.

[24] Zhou Y., Mu W., Zhang J., Wen S.W., Pakhale S. (2022). Global prevalence of non-tuberculous mycobacteria in adults with non-cystic fibrosis bronchiectasis 2006–2021: a systematic review and meta-analysis.. BMJ Open.

[25] Zhu Y., Xie J., He X., Peng B., Wang C., Zhang G., Xu J., Gao Y. (2021). Prevalence and clinical characteristics of nontuberculous mycobacteria in patients with bronchiectasis: A systematic review and meta-analysis.. Respiration.

[26] Yin H., Gu X., Wang Y., Fan G., Lu B., Liu M., Wang C., Cao B., Wang C. (2021). Clinical characteristics of patients with bronchiectasis with nontuberculous mycobacterial disease in Mainland China: a single center cross-sectional study.. BMC Infect. Dis..

[27] Wang H., Ji X.B., Mao B., Li C.W., Lu H.W., Xu J.F. (2018). *Pseudomonas aeruginosa* isolation in patients with non-cystic fibrosis bronchiectasis: A retrospective study.. BMJ Open.

[28] Shteinberg M., Stein N., Adir Y., Ken-Dror S., Shitrit D., Bendayan D., Fuks L., Saliba W. (2018). Prevalence, risk factors and prognosis of nontuberculous mycobacterial infection among people with bronchiectasis: a population survey.. Eur. Respir. J..

[29] Suska K., Amati F., Sotgiu G., Gramegna A., Mantero M., Ori M., Ferrarese M., Codecasa L.R., Stainer A., Blasi F., Aliberti S. (2022). Nontuberculous mycobacteria infection and pulmonary disease in bronchiectasis.. ERJ Open Res..

[30] Zheng M., Chen X., Chen Q., Chen X., Huang M. (2024). Employing multicolor melting curve analysis to rapidly identify non-tuberculous mycobacteria in patients with bronchiectasis: A study from a Pulmonary Hospital in the Fuzhou District of China, 2018-2022.. Crit. Rev. Immunol..

[31] Máiz L., Girón R., Olveira C., Vendrell M., Nieto R., Martínez-García M.A. (2016). Prevalence and factors associated with nontuberculous mycobacteria in non-cystic fibrosis bronchiectasis: a multicenter observational study.. BMC Infect. Dis..

[32] Faverio P., Stainer A., Bonaiti G., Zucchetti S., Simonetta E., Lapadula G., Marruchella A., Gori A., Blasi F., Codecasa L., Pesci A., Chalmers J., Loebinger M., Aliberti S. (2016). Characterizing non-tuberculous mycobacteria infection in bronchiectasis.. Int. J. Mol. Sci..

[33] Mirsaeidi M., Hadid W., Ericsoussi B., Rodgers D., Sadikot R.T. (2013). Non-tuberculous mycobacterial disease is common in patients with non-cystic fibrosis bronchiectasis.. Int. J. Infect. Dis..

[34] Pham H.V., Vu T.T., Bui N.T.T., Le A.L.T. (2023). Characterization of Nontuberculous Mycobacterium species detected from sputum samples of subjects in northern Vietnam.. Southeast Asian J. Trop. Med. Public Health.

[35] Bonnet M., San K.C., Pho Y., Sok C., Dousset J.P., Brant W., Hurtado N., Eam K.K., Ardizzoni E., Heng S., Godreuil S., Yew W.W., Hewison C. (2017). Nontuberculous Mycobacteria infections at a provincial reference Hospital, Cambodia.. Emerg. Infect. Dis..

[36] Angrill J., Agustí C., de Celis R., Rañó A., Gonzalez J., Solé T., Xaubet A., Rodriguez-Roisin R., Torres A. (2002). Bacterial colonisation in patients with bronchiectasis: Microbiological pattern and risk factors.. Thorax.

[37] Yang B., Kim T., Ryu J., Park H., Hwangbo B., Kong S.Y., Kwon Y.S., Lee S., Ra S., Oh Y.M., Sohn J., Choe K., Choi H., Lee H. (2021). Increased incidence and associated risk factors of aspergillosis in patients with bronchiectasis.. J. Pers. Med..

[38] Cheng W.C., Chang C.L., Sheu C.C., Wang P.H., Hsieh M., Chen M.T., Ou W.F., Wei Y.F., Yang T.M., Lan C.C., Wang C.Y., Lin C.B., Lin M.S., Wang Y.T., Lin C.H., Liu S.F., Cheng M.H., Chen Y.F., Peng C.K., Chan M.C., Chen C.Y., Jao L.Y., Wang Y.H., Chen C.J., Chen S.P., Tsai Y.H., Cheng S.L., Lin H.C., Chien J.Y., Wang H.C., Hsu W.H., Taiwan Bronchiectasis Research Collaboration (TBARC) (2024). Correlating Reiff scores with clinical, functional, and prognostic factors: Characterizing noncystic fibrosis bronchiectasis severity: Validation from a nationwide multicenter study in Taiwan.. Eur. J. Med. Res..

