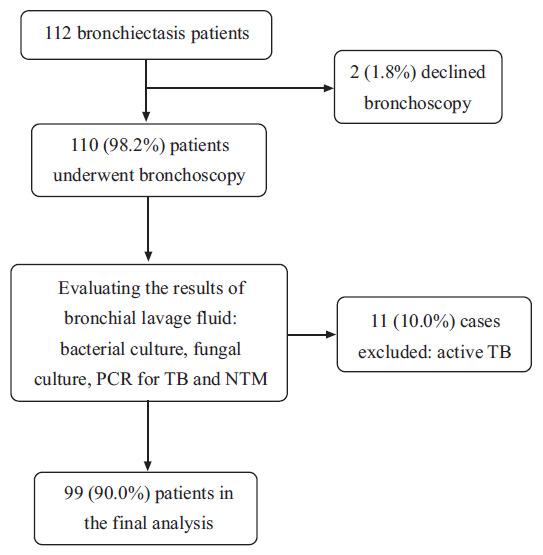# Corrigendum to: Potential Microorganisms from Bronchial Lavage Fluid in Bronchiectasis Patients: Bacteria, Nontuberculous Mycobacteria, and Fungi

**DOI:** 10.2174/0118743064392945250612511121

**Published:** 2025-12-30

**Authors:** Lam Nguyen Ho, Quoc-Khanh Tran-Le, Hoang Kim Tu Trinh, Vu Le-Thuong, Van Pham-Hung, Huong Pham-Thien, Phu Truong-Thien, Thong Dang-Vu, Dung Lam-Quoc, Ngoc Tran-Van

**Affiliations:** 1 Department of Internal Medicine, University of Medicine and Pharmacy at Ho Chi Minh City, Vietnam; 2 Respiratory Department, University Medical Center Ho Chi Minh City, Ho Chi Minh City, Vietnam; 3 Respiratory department, Cho Ray’s hospital, Vietnam; 4 Center for Molecular Biomedicine, University of Medicine and Pharmacy at Ho Chi Minh City, Vietnam; 5 Ngoc Minh Clinic, Ho Chi Minh City, Vietnam; 6 Microbiology, International Research Institute of Gene and Immunology, Ho Chi Minh City, Vietnam; 7 Microbiology, Phan Chau Trinh University, Vietnam; 8 Microbiology department, Cho Ray’s hospital, Ho Chi Minh City, Vietnam

The author has requested corrections to the corresponding author’s email address and the spelling in Figure 1 in their article titled “Potential Microorganisms from Bronchial Lavage Fluid in Bronchiectasis Patients: Bacteria, Nontuberculous Mycobacteria, and Fungi” published in “The Open Respiratory Medicine Journal,” 2025, 19, e18743064392945 [1].

We apologize for any inconvenience caused and appreciate the opportunity to rectify this matter.

## The original article can be found online at

https://openrespiratorymedicinejournal.com/VOLUME/19/ELOCATOR/e18743064392945/FULLTEXT/

## Original:

Corresponding Email Address:

E-mail: bsholam1986@gmail.com

## Corrected:

Corresponding Email Address:

E-mails: ***lam.nh@umc.edu.vn***, ***nguyenholam@ump.edu.vn*** and ***bsholam1986@gmail.com***

## Original:

**Figure 1:**

**Figure:**
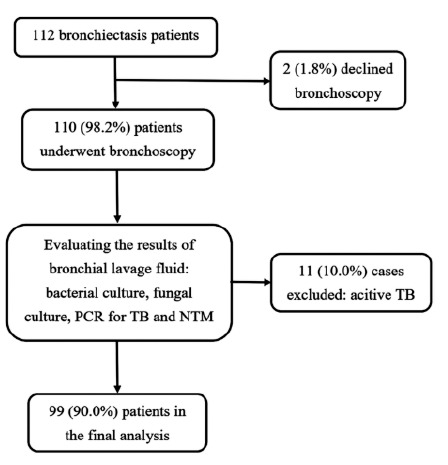


## Corrected:

**Figure 1:**

Spelling of active has been rectified.

**Figure:**