# Supplementary material for: Potential Microorganisms from Bronchial Lavage Fluid in Bronchiectasis Patients: Bacteria, Nontuberculous Mycobacteria, and Fungi
Source: Open Respir Med J. 2025 Jun 18;19:e18743064392945. doi: 10.2174/0118743064392945250613055623 (PMC12481589; doi:10.2174/0118743064392945250613055623)
Supplement: Supplementary file 1 [file TORMJ-19-E18743064392945_SD1.pdf]

The Open Respiratory Medicine Journal
DOI: 10.2174/0118743064392945250613055623, 2025, 19, e18743064392945
ISSN: 1874-3064

RESEARCH ARTICLE
OPEN ACCESS

Potential Microorganisms from Bronchial Lavage Fluid in Bronchiectasis Patients: Bacteria, Nontuberculous Mycobacteria, and Fungi

Lam Nguyen Ho[1,2,3,5,*], Quoc-Khanh Tran-Le[1], Hoang Kim Tu Trinh[4], Vu Le-Thuong[1,2], Van Pham-Hung[6,7], Huong Pham-Thien[6], Phu Truong-Thien[8], Thong Dang-Vu[3], Dung Lam-Quoc[3] and Ngoc Tran-Van[1,5]

[1]*Department of Internal Medicine, University of Medicine and Pharmacy at Ho Chi Minh City, Vietnam*
[2]*Department of Respiratory , University Medical Center Ho Chi Minh City, Ho Chi Minh City, Vietnam*
[3]*Department of Respiratory , Cho Ray's Hospital, Vietnam*
[4]*Center for Molecular Biomedicine, University of Medicine and Pharmacy at Ho Chi Minh City, Vietnam*
[5]*Ngoc Minh Clinic, Ho Chi Minh City, Vietnam*
[6]*Microbiology, International Research Institute of Gene and Immunology, Ho Chi Minh City, Vietnam*
[7]*Microbiology, Phan Chau Trinh University, Vietnam*
[8]*Department of Microbiology , Cho Ray's hospital, Ho Chi Minh City, Vietnam*

Abstract:

Introduction: Bronchiectasis is a chronic lung disease characterized by irreversible bronchial dilation, often accompanied by persistent infections. Compared to sputum, the microbiological results of bronchial lavage fluid (BLF) from stable bronchiectasis patients are typically less explored. There is emerging evidence on the role of non-tuberculous mycobacteria (NTM) in the progression of bronchiectasis. This study aims to investigate the microbiological profiles of BLF and the rate of NTM detection in stable bronchiectasis patients.

Methods: We conducted a prospective observational multicenter study at two endoscopy units of Cho Ray's Hospital and University Medical Center Ho Chi Minh City, from January 2023 to February 2024. Adult patients with bronchiectasis who underwent bronchoscopy were enrolled, and the BLF was collected. The BLF samples were analyzed for bacterial and fungal pathogens using culture methods, and for NTM using the multiplex polymerase chain reaction (PCR) technique.

Results: Of the 112 initially assessed patients, 99 were eligible for this study. The mean age was 63 years, and 55.6% were female. Bacterial cultures were positive in 41.9% of cases (36/86), predominantly with isolates of *Klebsiella pneumoniae* and *Pseudomonas aeruginosa*. Multi-drug resistant (MDR) *K. pneumoniae* and *Acinetobacter baumannii* were notably detected. Using PCR, NTM was detected in 52.5% of patients (52/99), predominantly slow-growing species such as *Mycobacterium xenopi* and *Mycobacterium avium-intracellulare* complex. Fungal cultures were positive in 24.6% of cases (17/69), primarily involving *Candida* spp. and *Aspergillus* spp. Patients with higher bronchiectasis severity index had higher rates of positive bacterial culture, but lower rates of NTM detection.

Conclusion: This study demonstrated a microbial diversity in BLF, notably NTM and MDR bacteria in Vietnamese patients with bronchiectasis, emphasizing the need for routine, comprehensive microbial assessment for bronchiectasis patients. The incorporation of advanced molecular techniques can improve the detection of NTM in these patients.

Keywords: Bacteria, Bronchiectasis, Bronchial lavage fluid, Fungi, Nontuberculous mycobacteria, Pathogen.

© 2025 The Author(s). Published by Bentham Open.
This is an open access article distributed under the terms of the Creative Commons Attribution 4.0 International Public License (CC-BY 4.0), a copy of which is available at: https://creativecommons.org/licenses/by/4.0/legalcode. This license permits unrestricted use, distribution, and reproduction in any medium, provided the original author and source are credited.

Received: February 21, 2025
Revised: April 30, 2025
Accepted: May 07, 2025
Published: June 18, 2025

*Address correspondence to this author ait the Department of Respiratory, University Medical Center Ho Chi Minh City, 215, Hong Bang, Ward 11, District 5, Ho Chi Minh City, Vietnam; E-mails: lam.nh@umc.edu.vn, nguyenholam@ump.edu.vn, bsholam1986@gmail.com

Cite as: Ho L, Tran-Le Q, Trinh H, Le-Thuong V, Pham-Hung V, Pham-Thien H, Truong-Thien P, Dang-Vu T, Lam-Quoc D, Tran-Van N. Potential Microorganisms from Bronchial Lavage Fluid in Bronchiectasis Patients: Bacteria, Nontuberculous Mycobacteria, and Fungi. Open Respir Med J, 2025; 19: e18743064392945.
http://dx.doi.org/10.2174/0118743064392945250613055623

Send Orders for Reprints to reprints@benthamscience.net

Supplement S1. **Principles of designing primers and Taqman probes in multiplex real-time PCR:**

[1] Primers must have a melting temperature of 60°C and a matching efficiency on the target DNA of at least 75%.
[2] Primers should be at least 18 bases long and no longer than 30 bases.
[3] Primers should not have more than 3 identical bases at both ends.
[4] Primers should not self-pair. Forward and reverse primers should not have sequences that allow them to bind to each other. primers also must not have self-complementary regions to form hairpin structures.
[5] Taqman probes must have a melting temperature of 70°C and a matching efficiency on the target DNA of at least 75%.
[6] Taqman probes should be at least 20 bases long and no longer than 29 bases.
[7] Taqman probes should not have a 5' end with G, as G adsorbs the fluorescence of the reporter attached to the 5' end of the Taqman probe.
[8] Taqman probes should not have more than 3 identical bases at both ends.
[9] Taqman probes should not self-pair, pair with primers, or form hairpin structures.
[10] In multiplex real-time PCR, primers and Taqman probes should be designed to avoid pairing with each other.
[11] In multiplex real-time PCR, the emission wavelengths of reporters should be separated to prevent cross-signal detection.

Supplement Table S1. 5'-3' Primer and Probe Sequences for MPL Real-Time PCR Detection of Mycobacteria Species

MPL-rPCR	Target Species	Fluorophore	Forward Primer Sequence (5'-3')	Reverse Primer Sequence (5'-3')
MPL-rPCR 1	M. tuberculosis/M. bovis	FAM	TGCAAGTCGAACGGAAAGGT	TCACGAACAACGCGACAAAC
	M. avium	HEX	TCGAACGGAAAGGCCTCTTC	TCACGAACAACGCGACAAAC
	M. ulcerans/M. marinum	TexasRED	ACTGAGATACGGCCCAGACT	TCACGAACAACGCGACAAAC
	M. intracellulare	CY5	AATTCCTGGTGTAGCGGTGG	TAGCATGTGTGAAGCCCTGG
MPL-rPCR 2	M. kansasii/gastri	FAM	ACTGAGATACGGCCCAGACT	TCACGAACAACGCGACAAAC
	M. scrofulaceum/kansasii/simiae/gastri	HEX	TGGTAGTCCACGCCGTAAAC	CACCTTCCTCCGAGTTGACC
	M. xenopi	TexasRED	ACGCGAAGAACCTTACCTGG	TAGCATGTGTGAAGCCCTGG
	M. fortuitum	CY5	CTGCCCTGCACTTTGGGATA	AGTCTGGGCCGTATCTCAGT
MPL-rPCR 3	M. senegalense	FAM	GCAGGGGAGACTGGAATTCC	TGTGCATGTCAAACCCAGGT
	M. abscessus/chelonae	HEX	ACCTGGGTTTGACATGCACA	CGGCCATTGTAGCATGTGTG
	M. genavense	TexasRED	TCCTTCCTTGGAATCCGTGC	TAGCATGTGTGAAGCCCTGG
	M. gordonae	CY5	CACTGGGACTGAGATACGGC	CGACAAACCACCTACGAGCT
MPL-rPCR 4	M. malmoense	FAM	TGCACTTCGGGATAAGCCTG	AGAAAACCCGGACCTTCGTC
	M. tilburgii	HEX	CACTGGGACTGAGATACGGC	CCACCGCTACACCAGGAATT
	M. smegmatis	TexasRED	ACCTGGGTTTGACATGCACA	CACCTTCCTCCGAGTTGACC
	M. szulgai	CY5	GCAGGGGAGACTGGAATTCC	CCAGGTAAGGTTCTTCGCGT
MPL-rPCR 5	M. leprae	FAM	AGCTCGTAGGTGGTTTGTCG	GTTTACGGCGTGGACTACCA
	M. flavescens/smegmatis	HEX	ACCTCTTTCGGTAGGGACGA	GGAATTCCAGTCTCCCCTGC
	M. terrae	TexasRED	GTCGAACGGAAAGGCTTTCG	AGTCTGGGCCGTATCTCAGT
	M. cosmeticum	CY5	ATCAGTTCCCTTGTGGCCTG	CGGCCATTGTAGCATGTGTG

Supplement Table S2. . Antimicrobial resistance profiles of bacteria isolated from bronchial lavage fluid

	Carbapenems	Cefepime	Cephalosporins 3	Fluoroquinolones	Aminoglycosides	TMP/SMX
**Klebsiella pneumoniae** (n = 11)	3/11	0/8	2/11	5/11	2/11	1/11
**Pseudomonas aeruginosa** (n = 10)	0/10	0/10	1/9	1/10	0/10	NA
**Haemophilus influenzae** (n = 5)	0/3	0/1	0/3	1/4	NA	3/3
**Acinetobacter baumannii** (n = 4)	4/4	4/4	4/4	4/4	3/4	2/4
**Staphylococcus aureus** (n = 2)	NA	NA	NA	NA	0/2	0/2
**Escherichia coli** (n = 2)	0/2	NA	2/2	1/2	2/2	2/2

***Non-susceptible to at least 1 antibiotic of the corresponding antibiotic class**
Carbapenems: Meropenem, Imipenem, Ertapenem
Cephalosporins 3: Ceftazidime, Ceftriaxone, Cefotaxime
Fluroquinolones: Levofloxacin, Ciprofloxacin
Aminoglycosides: Amikacin, Gentamycin, Tobramycin
TMP/SMX: Trimethoprim - Sulfamethoxazole **Intermediate susceptibility is classified as non-susceptible**
NA: not applicable
